# Case of Hon. Charles Sumner

**Published:** 1857-02

**Authors:** Marshall S. Perry

**Affiliations:** Boston


"*Case of* Hon. CHARLES SUMNER. Read before the Boston Society, for Medical Improvement. By MARSHALL S. PERRY, M. D., Boston."

Under the above title, we find, in a late number of the Boston Medical & Surgical Journal, what professes to be a *scientific* account of the case of Hon. Charles Sumner, for the *improvement* of the members of the Boston Medical Society. After stating what was very important to the advance of medical knowledge, that the *"*assault* was made upon Mr. Sumner, in the *Senate* of the United States, on Thursday, May 22d," (which, by the way, is not true, unless the Senate Chamber constitutes the Senate,) we have a very confused and unsatisfactory account of the injuries sustained, as an example of which, it is stated that "the gashes went through the scalp to the bone, which was laid bare, but it is *supposed* not fractured."

*The italics are ours.

It seems to us, (though we do not profess to be much of a surgeon,) that it ought to have been determined whether there was *really* any fracture of the scull, in so important a case as that of the Hon. Charles Sumner, whose injuries have been called by Dr. Wendell Holmes, (we believe,) "the wounds of the nation."

It seems that he remained for some time in Washington city, and next, in the progress of this remarkable case, he turns up in Philadelphia, where, under the care of Dr. Wistar, he was found to be in "a state of extreme nervous exhaustion, (very natural result of fright, almost unto death,) "but no evidence of organic disease;" and we then here of him on the Alleghany Mountains, in Pennsylvania, where, under the influence of mountain air, mountain water, and change of climate, there was a gradual stringing up and intonation of the whole body; until, in about five weeks, the improvement was so great "that Mr. Sumner could not be *persuaded* that he was still an invalid." He finally returned to Boston, where it seems that he has gradually recovered his appetite, "*and sleeps much better than he did*;" though with all his travels and advisers, the *scientific* reporter naively acknowledges that "it is impossible to decide, with absolute certainty, what the pathological condition of Mr. Sumner's brain has been," and winds up with the sapient conclusion, that "*had the patient died, a post mortem examination would have determined conclusively, the character of the injury.*"

As it might not be known generally, we will state that this is the report of the physician who was called all the way from Boston, to attend the individual, whose condition he professes to describe.

We must express our regret that such an article should have appeared in so respectable a journal; and particularly that the editor should have called special attention to it, when, from his own acknowledgement, "notwithstanding the careful observation of the patient, by eminent medical men, it is difficult to say what is the exact nature of the lesion;" the legitimate deduction from which, we have a right to conclude, from all the circumstances, is, that it has been a case manufactured for the occasion.

We do not know that this matter would have been noticed

(having become so much accustomed to similar exhibitions of fanatical influence), but for the frequent protests from Northern journals, &c., against what they term "Sectional Medicine."

What particle of scientific information is conveyed by the report of the above case, we are utterly at a loss to perceive; and it seems to us to come with a bad grace, from those who are constantly expressing opposition to the introduction of political matters into scientific discussions; who even protest against the effort upon the part of Southern Editors and Southern Colleges, to build up Southern medicine, by the patronage which *belongs to the soil.*

It is our intention to use whatever influence we may possess, for the up-building of the South, in every particular; for the advancement of all the interests, whether political, commercial or educational, and to this end we shall especially advocate the education of Southern men, upon Southern soil; and an entire disruption of the cords of vassalage which have so long bound us hand and foot to the North—if this be "*Sectional Medicine*," then are we, its advocates.

If the most liberal and generous spirit in every particular, had been manifested upon the part of the North, towards our section of the Union, the most ordinary regard for the prosperity and independence of the region in which we make our homes, it seems to us, should prompt the advocacy of such views; and when we have a large majority of the whole people of the Northern portion of the confederacy prepared to upturn our very foundations, it is time, we think, to begin to take care of ourselves, and at least cease to add to the strength and power of those, who need but little more, to become our oppressors in, or to force us out of, the Union.

